# Running-Related Injury Incidence: Does It Correlate with Kinematic Sub-groups of Runners? A Scoping Review

**DOI:** 10.1007/s40279-023-01984-0

**Published:** 2024-01-27

**Authors:** Léa Adamson, Liam Vandamme, Trevor Prior, Stuart Charles Miller

**Affiliations:** 1 School of Medicine, Sir Alexander Fleming Building, Imperial College London, London, UK; 2 Sports and Exercise Medicine, William Harvey Research Institute, Queen Mary University of London, London, UK; 3 Digital Environment Research Institute (DERI), Queen Mary University of London, London, UK

## Abstract

**Background:**

Historically, kinematic measures have been compared across injured and non-injured groups of runners, failing to take into account variability in kinematic patterns that exist independent of injury, and resulting in false positives. Research led by gait patterns and not pre-defined injury status is called for, to better understand running-related injury (RRI) aetiology and within- and between-group variability.

**Objectives:**

Synthesise evidence for the existence of distinct kinematic sub-groups across a population of injured and healthy runners and assess between-group variability in kinematics, demographics and injury incidence.

**Data Sources:**

Electronic database search: PubMed, Web of Science, Cochrane Central Register of Controlled Trials (Wiley), Embase, OVID, Scopus.

**Eligibility Criteria:**

Original, peer-reviewed, research articles, published from database start to August 2022 and limited to English language were searched for quantitative and mixed-methods full-text studies that clustered injured runners according to kinematic variables.

**Results:**

Five studies (*n* = 690) were included in the review. All studies detected the presence of distinct kinematic sub-groups of runners through cluster analysis. Sub-groups were defined by multiple differences in hip, knee and foot kinematics. Sex, step rate and running speed also varied significantly between groups. Random injury dispersal across sub-groups suggests no strong evidence for an association between kinematic sub-groups and injury type or location.

**Conclusion:**

Sub-groups containing homogeneous gait patterns exist across healthy and injured populations of runners. It is likely that a single injury may be represented by multiple movement patterns, and therefore kinematics may not predict injury risk. Research to better understand the underlying causes of kinematic variability, and their associations with RRI, is warranted.

**Supplementary Information:**

The online version contains supplementary material available at 10.1007/s40279-023-01984-0.

## Key Points

**Table:** 

Homogeneous sub-groups may be identified within an injured population of runners, differentiated by a number of kinematic and functional characteristics.
For any given kinematic sub-group, there is no significant increased risk of running-related injury, by either diagnosis or location.
No consistent association between movement patterns and RRI has been clearly shown.

## Introduction

Running is increasingly highlighted to be one of the most popular recreational and competitive sports, with multiple well-documented public health benefits [1]. Within England alone, an estimated 3.7 million [2] run each month, augmented further by the Covid-19 lockdown and changes in exercise behaviour to favour outdoor, solo activities [3]. Despite its popularity, a running-related injury (RRI) remains a major barrier to participation for all abilities, with reported annual incidence ranging from 19.4 to 79.3% [4]. A general consensus is that 50% of runners will experience an RRI annually [5].

It is unsurprising, therefore, that a large body of research has been undertaken to better understand risk factors for RRI development. In this respect, multiple aetiological frameworks have been posited, with abnormal biomechanics thought to contribute to injury through cumulative structure-specific loading [6, 7]. Attempts to better understand RRI pathomechanics have been made within numerous studies undertaking gait analysis. Traditionally, these studies group individual participants according to injury and compare multiple kinematic variables across injured and uninjured groups. In this way, a number of injury-specific biomechanical factors associated with RRI have been proposed. For example, increased peak hip abduction angles have been associated with iliotibial band syndrome [8] and medial tibial stress [9], and reduced knee flexion observed in Achille’s tendinopathy [10] and patellofemoral pain (PFPS) [11].

This method of grouping individuals in accordance with a pre-defined injury status has its limitations. First, retrospective comparison of multiple variables both increases the probability of false positives [12] and fails to distinguish causality from compensatory mechanisms to injury. Second, studies have revealed the existence of distinct kinematic sub-groups (i.e. groups of runners who have similar gait kinematics) within both injured and healthy populations [13–15]. Taking into account this variability, it would therefore be incorrect to assume that all individuals with the same RRI exhibit the same highly specific kinematic variables. As such, the current practice of grouping based on injury, and then running multiple comparisons, has potentially led to a misunderstanding about the relationship between gait and injury.

To address these limitations, machine learning techniques, such as hierarchical cluster analysis (HCA), are increasingly being adopted to define groups of individuals based on similarities in movement patterns. This method of analysis allows us to objectively determine whether specific injuries correlate more closely with certain sub-groups, and thus whether individuals with the same injury move in the same way.

The aims of this scoping review were to: (i) synthesise current evidence for the existence of homogeneous kinematic sub-groups within an injured population; (ii) compare kinematic characteristics of sub-groups; (iii) observe within- and between-group variability in RRI incidence; (iv) assess for differences in non-kinematic variables between clusters; and (v) describe knowledge gaps in the literature and highlight potential areas for future research.

## Methods

### Protocol

The Preferred Reporting Items for Systematic Reviews and Meta-analysis scoping reviews (PRISMA-ScR) guidelines [16] were followed. The protocol for this scoping review has not previously been published.

### Eligibility Criteria

To qualify for inclusion in the review, articles must have clustered individuals according to running kinematics and not pre-determined injury status. Therefore, any study that takes on a supervised approach (i.e. groups participants on the basis of injury before running group comparison on kinematics) would not be included. Peer-reviewed journal articles and theses were included if they were: available in English language, exclusive to humans and focused on overuse injuries. All primary research in the form of quantitative and mixed-methods analyses was included. Articles that clustered gait patterns according to injury were excluded, as were review articles, case reports, paediatric, animal and in vitro studies, and those observing only healthy populations.

### Information Sources

A comprehensive database search was conducted on 23 November 2021 (and updated on 19 August 2022) and applied to the following six electronic databases: PubMed, Web of Science, Cochrane Central Register of Controlled Trials (Wiley), Embase, OVID and Scopus. The search strategy was devised following discussion amongst the research team, and further refined upon reviewing of literature in an iterative process. The NLM Medical Subject Headings (MeSH) thesaurus was used to identify keywords, which were incorporated into the final search strategy. All potential references were imported into Covidence systematic review software (Veritas Health Innovation, Melbourne, Australia) for duplicate removal and screening. The reference list, and citations, of all included papers and any relevant reviews were further screened. Finally, all included papers were searched within the Connectedpapers.com [17] online application.

### Search Strategy

The search strategy was broken up into four components (biomechanics, clustering, injury and movement). Search terms were developed under each component iteratively. The final main search is in Table 1.

**Table 1:** Database search strategy and results. Topics were separated by ‘AND’ and search terms separated by ‘OR’

Keyword	Search term
Biomechanics	biomechanic* or kinematic* or "motion capture" or video
Clustering	unsupervised or cluster* or variability or nonlinear* or non-linear* or PCA or "principle component analysis" or dynamic*
Injury	injur* or dysfunction* or dysfunction
Movement	gait or runn* or run or jog or jogg*

Additional filters for Scopus and OVID included limiting search to title, abstract and keyword

### Selection of Sources of Evidence

All publications underwent blind review by two independent reviewers (L.A., L.V. or S.M.). Reviewers sequentially reviewed the titles and abstracts of all publications retrieved by the database search after duplicate removal. Potentially relevant publications had their full texts retrieved and screened by two independent reviewers. Any conflicts were resolved by a group discussion to decide the final included studies.

### Data Charting Process

Comprehensive data charting tables were developed to extract and summarise information on study population, methodology and results. Only information of relevance was included, with data deemed irrelevant to the aims and objectives of the review omitted from results. Team-based discussions were then undertaken to verify data and resolve any inconsistencies.

### Data Items

Information was extracted on (i) study characteristics (primary author, year of publication, country of origin, funder), (ii) population characteristics (sample size, height, mass, running experience), (iii) injury characteristics (diagnosis, definition, symptom duration), (iv) methodology (study conditions, data collection methods), (v) outcome measures and (vi) covariates. This information was illustrated in tabular format, separating population demographics from study characteristics and methodology.

Outcome measures of interest were kinematic variables in all three planes (frontal, sagittal and transverse) for the pelvis, hip, knee, ankle and foot (i.e. lower limb). As specific variables reported varied across studies, all relevant variables were included in this scoping review. All descriptive analyses were conducted using Microsoft Excel (Version 16.43; Microsoft Corp., Redmond, WA, USA) and cross-checked within the research team.

### Critical Appraisal of Sources of Evidence

The methodological quality of included papers was critically assessed by two reviewers using a modified Downs and Black checklist. Modifications were made to remove questions relating to interventions due to the observational nature of all included studies. Upon completion, the maximum total score was deemed to be 15, from a total of 14 screening questions. Group agreement within the research team determined further risk of bias (ROB) assessment to be unnecessary due to both the observational nature of the included publications and the nature of this scoping review.

### Synthesis of Results

Synthesis of results was mainly done narratively. Relevant results, *p*-values and effect sizes (Cohen’s *d*) are displayed in tabular format for ease of comparison. Table headings assessed (i) cluster analysis methods, (ii) homogeneous kinematic sub-groups identified, (iii) within-group kinematic characteristics, (iv) RRI incidence within sub-groups by diagnosis and (v) RRI incidence within sub-groups by location. Any covariates such as demographic, running volume, speed and experience that were reported within the studies are also reported.

### Additional Analyses

Due to the heterogeneity of methodological approaches utilised across the included studies, the heterogeneity in biomechanical variables, and the nature of this scoping review, no additional analyses were performed.

## Results

### Selection of Sources of Evidence

**Fig. 1:**
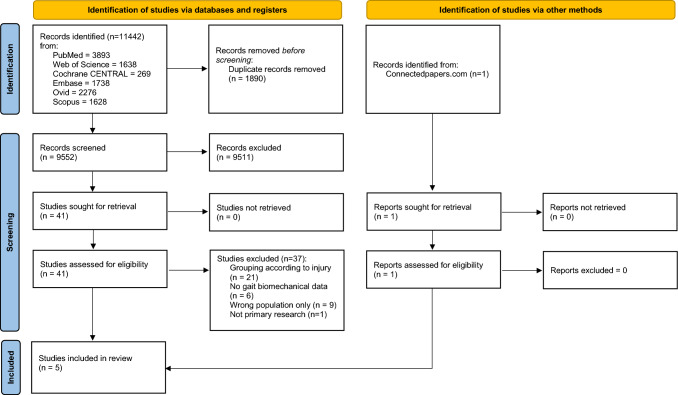
PRISMA flowchart showing the screening process undertaken within the scoping review

Following duplicate removal, 9552 studies were screened. Following full screening, four studies were included. A further study was found following screening of references and connectedpapers.com (Fig. 1).

### Characteristics of Sources of Evidence

Out of the five included studies, four were primary and from peer-reviewed literature, and one was grey literature. All of the primary studies had a cross-sectional case–control design, and the majority were retrospective. Martin et al. [18] was the only study to observe injury incidence from prospectively collected healthy kinematic data. The grey literature included was a recent master’s thesis [19], which was sourced from their university repository and had not been (peer-reviewed) published prior to the completion of this scoping review. Three studies [13, 18, 20] included both injured and healthy runners in their populations, and the other two [19, 21] observed solely injured groups.

Study characteristics are detailed in Table 2. All included studies shared a similar primary focus, utilising unsupervised clustering techniques (*K*-means or hierarchical) to identify kinematic sub-groups of runners. Some studies employed a principal component analysis (PCA) to either reduce multi-collinearity between variables [13, 20] or to determine variability in outcome measures [21]. Studies then compared intra- and inter-group kinematic characteristics and injury prevalence. Watari et al. [21] and Martin et al. [18] focused solely on runners with PFPS and bony stress injury (BSI), respectively, whilst Dingenen et al. [20], Jauhiainen et al. [13] and Gore [19] observed a number of different RRIs.

**Table 2:** Study characteristics of included studies

First author, year	Country, cohort	Sample size, *N*	Running population	RRI(s)	Injury incidence	Injury definition	Symptom duration (weeks)	Data collection procedure	Preliminary analysis	Kinematic outcome variables
Dingenen et al. 2020 [20]	Belgium, Australia HU, PR	53 (15 M, 38 F)	Recreational	PFPS, ITB, AT, PF, MTSS, non-specific muscle sprains, tendinopathies	Medically diagnosed RRI (*n* = 53)	Running-related LL MSK pain causing a restriction/cessation of running (distance, speed, duration, training) > 7 days or 3 consecutive training sessions, or that requires consultation with a health professional	> 1	Instrumented treadmill run, self-selected speed 2D motion capture Regular running shoes	7 steps analysed visually by blinded research Bilateral	Frontal and sagittal trunk, hip/pelvis, knee and ankle/foot joint angles Midstance and initial contact
Watari et al. 2018 [21]	Canada RIC, UC	110 (44 M, 66 F)	Physically active Recreational	PFPS	Medically diagnosed RRI (*n* = 110)	Insidious onset, non-traumatic anterior knee pain Induced and exacerbated by: physical activity, squatting, sitting, stair ascent/descent TOP of patellar facets	> 4	Instrumented treadmill run, self-selected speed 3D motion capture Nike Pegasus	60–80 steps analysed Each step time-normalised × 100 (80 stance, 20 swing) Bilateral	Frontal, sagittal and transverse hip/pelvis, knee and ankle joint angles Pelvic acceleration patterns
Jauhiainen et al. 2020 [13]	Finland, Canada RIC, UC	291 (146 F, 145 M)	Competitive (*n* = 57) Recreational (*n* = 234)	PFPS, ITB, AT, PF, MTSS Non-specific muscle sprains, tendinopathies	Medically diagnosed RRI (*n* = 266) Uninjured (*n* = 25)	Running-related LL MSK pain causing a restriction/cessation of running (distance, speed, duration, training) > 7 days or 3 consecutive training sessions, or that requires consultation with a health professional	> 1	Instrumented treadmill run, self-selected speed 3D motion capture Nike Pegasus	30–40 steps analysed Each step time-normalised × 100 (65 stance, 35 swing) Bilateral	Frontal, sagittal and transverse hip/pelvis, thigh, shank, knee and ankle/foot joint angles
Martin et al. 2022 [18]	USA UW, BAPD	53 (32 F, 21 M)	Collegiate-level competitive runners	BSI	Medically diagnosed RRI (*n* = 24) Uninjured (n = 29)	Stress fracture or bony reaction confirmed by presence of periosteal, marrow ± cortical oedema, as visualised on MRI	Not specified	Instrumented treadmill run at 4.47 m/s for 15 s after 30 s acclimation 3D motion capture Shoe type not specified	Joint angles estimated using standard inverse analyses from data from 14 body segments, scaled according to height, mass and segment length	Frontal, sagittal and transverse hip flexion and adduction, knee flexion, ankle dorsiflexion, split into strides (swing and stance phases) based on foot contact timing
Gore 2020 [19]	Ireland RISC	282	Novice and recreational runners	Not specified	RRI (*n* = 186) Uninjured (*n* = 96)	Running-related LL MSK pain causing a restriction/cessation of running (distance, speed, duration, training) > 7 days or 3 consecutive training sessions, or that requires consultation with a health professional	> 1	Instrumented treadmill run at 9 km/h for 1 min 3D motion capture with 16 Vicon cameras at 200 Hz Shoe type not specified	Events detected and steps extracted, time normalised to 101 points Bilateral	Foot strike patterns: maximum foot acceleration, mean foot acceleration from 6 to 20% of the foot strike, foot acceleration variation, maximum foot velocity, median foot velocity

*ITB* iliotibial band injury, *PFPS* patellofemoral pain syndrome, *AT* Achilles tendinopathy, *PT* patellar tendinopathy, *MTSS* medial tibial stress syndrome

Sample characteristics are described in Table 3. Where reported, demographics were similar across the groups for age, height, mass and running experience.

**Table 3:** Population characteristics of included studies (mean ± standard deviation)

First author, year	Sample size, *N*	*N* (males)	*N* (females)	Age	Height (cm)	Mass (kg)	Running experience (years)	Running speed (m/s)	Running step rate (steps/min)
Dingenen et al. 2020 [20]	53	15	38	31.3 ± 6.8	169.9 ± 7.8	65.6 ± 10.1	9.7 ± 8.4	2.75 ± 0.36	166.3 ± 8.3
Watari et al. 2018 [21]	110	44	66	34.6 ± 2.7	171.2 ± 6.5	67.9 ± 7.9	8.4 ± 7.7	2.61 ± 0.052	–
Jauhiainen et al. 2020 [13]	291	146	145	39.5 ± 11.2	172.1 ± 11.2	70.9 ± 13.4	–	2.51 ± 5.84	–
Martin et al. 2022 [18]	53	21	32	–	171 ± 10	61.3 ± 8.2	–	4.47	–
Gore 2022 [19]	282	–	–	–	–	–	–	2.5	–

– Data not provided, *N* number, *cm* centimetre, *kg* kilograms, *m/s* metres per second

### Critical Appraisal of Sources of Evidence and Risk of Bias

All studies were assessed for quality using the modified Downs and Black Index [22]. All four peer-reviewed studies were deemed to be of sound methodological quality, in concordance with criteria detailed in Table 4. Omittance of demographic and confounding data and failure to meet subsequent follow-on criteria meant that the grey literature [19] scored poorly overall.

**Table 4:** Modified Downs and Black checklist for study quality assessment of included studies

Included studies	Criteria															% Total
	(1)	(2)	(3)	(5)	(6)	(7)	(10)	(11)	(12)	(16)	(18)	(20)	(25)	(27)	Total	
Dingenen et al. [20]	1	1	1	1	1	1	1	1	1	1	1	1	1	1	14	93
Watari et al. [21]	1	1	1	2	1	1	1	1	1	1	1	1	1	1	15	100
Jauhiainen et al. [13]	1	1	1	1	1	1	1	1	1	1	1	1	1	1	14	93
Martin et al. [18]	1	1	1	1	1	1	0	1	1	1	1	1	1	0	12	80
Gore [19]	1	1	0	0	1	1	1	0	0	1	1	1	0	1	9	60

Scoring: ‘yes’ = 1, ‘no’ = 0, ‘unable to determine’ = U (scored as 0); ((5): ‘yes’ = 2, ‘partially’ = 1, ‘no’ = 0))

Criteria: (1) clear aim/hypothesis, (2) main outcome measures clearly described, (3) patient characteristics clearly described, (5) distribution of confounders described, (6) main finding clearly described, (7) random variability of main outcomes provided, (10) actual probability values reported, (11) subjects asked to participate representative of entire population, (12) subjects prepared to participate representative of entire population, (16) clear mentioning of data dredging (unplanned analysis), (18) appropriate statistical analysis, (20) valid and reliable outcome measures, (25) adequate adjustment for confounding, (27) did study have sufficient power to detect a clinically important effect

### Summary of Findings

All findings of relevance to the aims of this review, including *p*-values and effect sizes, are summarised in Table 5.

**Table 5:** Study results of included studies, with *p* values and effect sizes

First author, year	Statistical analysis	*n* sub-groups identified	Kinematic characteristics of sub-groups (s)	*p* value	Effect size	RRI incidence by location (% sub-group injured	RRI incidence by diagnosis (%)
Dingenen et al. 2020 [20]	*K*-means cluster analysis following PPMCC to reduce multi-collinearity between variables	2	*S1 (27 F, 12 M)*			*S1* ↑ LL (51.3%) *S2* ↑ Hip (35.7%) ↑ Foot (7.1%) ↔ Knee (36%) No significant differences found between sub-groups	*S1* ↑ PFPS (10.3) ↑ PT (2.6) ↑ MTSS (38.5) ↑ AT (5.1) *S2* ↑ GT (21.4) ↑ ITB (35.7) ↑ NSHP (7.1)
			↑ Foot inclination°—IC	*p* < 0.001	2.747		
			↑ Tibia inclination°—IC	*p* = 0.001	1.082		
			↑ Knee flexion°—MS	*p* = 0.085	0.549		
			*S2 (11 F, 3 M)*				
			↑ Hip adduction°—MS	*p* = 0.086	0.562		
Watari et al. 2018 [21]	HCA followed by PCA to determine variability in pelvic acceleration patterns Males and females analysed separately	2 (F) 1 (M)	*S1 (n = 26)*			n/a	n/a
			↑ Hip internal rotation° (M only)	*p* = 0.007	1.4		
			↓ Vertical displacement	*p* < 0.001	2.7		
			*S2 (n = 40)*				
			No significant characteristics				
			*M (n = 44)*				
			↑ Ankle eversion°	*p* < *0.001*	0.6		
			↓ Hip adduction°	*p* = 0.002	0.7		
			↓ Knee abduction°	*p* = 0.002	0.7		
			↓ Hip internal rotation° (M only)	*p* = 0.007	1.4		
Jauhiainen et al. 2020 [13]	HCA following PCA to reduce multi-collinearity between variables	5	*S1 (25 F, 70 M)*	*p* < 0.001	2.08	*S1 (88)* ↓ Hip/pelvis (12.6) ↑ Thigh (19) *S2 (90)* ↑ Ankle/foot (30) *S3 (100)* ↑ Knee (44) ↓ LL (9) *S4 (89)* ↓ Knee (14) *S5 (93)* ↑ LL (18) No significant differences found between sub-groups	n/a
			↓ Knee adduction°				
			↑ Knee flexion°				
			*S2 (38 F, 22 M)*	*p* < 0.001	2.05 (PC1)		
			↓ Knee flexion°		1.16 (PC2)		
			*S3 (11 F, 21 M)*				
			(↑ Hip adduction°, excursion and velocity)	*p* < 0.001	2.12		
			*S4 (13 F, 15 M)*		1.2 (PC3)		
			↑ Heel strike°		1.3 (PC4)		
			↑ Foot progression°	*p* < 0.001	1.04 (PC5)		
			*S5 (60 F, 16 M)*				
			↑ Rearfoot onset/offset eversion°				
			↓ Foot progression°				
			↑ Time to peak pronation	*p* < 0.001	1.10 (PC1)		
			↑ Hip adduction°, excursion°, velocity		1.17 (PC3)		
Martin et al. 2022 [18]	HCA following PCA to reduce multi-collinearity between variables Sub-groups determined using Ward algorithm	5	*S1 (3 F, 4 M)*		Not provided	*S1* (57) *S2* (57) *S3* (44) *S4* (42) *S5* (33) No significant differences found between sub-groups	
			Midfoot strike pattern				
			↑ Knee flexion°				
			↑ Knee extension moment				
			*S2 (3 F, 4 M)*				
			Forefoot strike pattern				
			↓ Knee flexion°	*p* < 0.0056			
			↑ Ankle plantarflexion moment				
			↓ Knee extension moment				
			*S3 (7 F, 11 M)*				
			↑ Peak hip extension moment				
			*S4 (10 F, 2 M)*				
			*S5 (9 F, 0 M)*				
			↓ Ankle plantarflexion moment				
Gore 2020 [19]	Comparison of 6 clustering models (*K*-means, hierarchical, OPTICS, mean shift, spectral and HDBSCANl) using one-way ANOVA Rand index to determine random assignments of injury to clusters	5	*S1*	*p* < 0.001	Not provided	n/a	n/a
			↓ % of time in ground contact				
			↑ Peak knee extension moment				
			*S2*				
			↑ Ankle dorsiflexion ° at FS	*p* < 0.006			
			↓ Peak hip extension moment	*p* < 0.001			
			↓ Peak knee extension moment				
			*S3*				
			*S4*				
			↓ Ankle dorsiflexion ° at FS	*p* < 0.001			
			*S5*				
			↑ % of time in ground contact	*p* < 0.001			
			↓ Peak vertical GRF				
			↓ Ankle plantarflexion moment				

*PPMCC* Pearson product-moment correlation coefficient, *IC* initial contact, *MS* midstance, *LL* lower limb, *ITB* iliotibial band injury, *PFPS* patellofemoral pain syndrome, *AT* Achilles tendinopathy, *PT* patellar tendinopathy, *MTSS* medial tibial stress syndrome, *GT* gluteal tendinopathy, *NSHP* non-specific hip pain, *FS* foot strike, *GRF* ground reaction force, ↑ highest recorded values, ↓ lowest recorded values

*Results lacking significance but included

### Synthesis of Results

#### Presence of Homogeneous Kinematic Sub-groups

All five studies identified distinct, homogeneous kinematic sub-groups of runners. Sub-group number varied from two to five. In general, there was a positive association between sample size and sub-group number, with the largest sample sizes seeming to differentiate into the most groups.

#### RRI Incidence within Sub-groups

There was very limited evidence to suggest that kinematic sub-groups differed in RRI incidence, regardless of the location or diagnosis. Although Dingenen et al. [20] suggested a higher proportion of lower-limb injuries in one group and hip injuries in another (two groups in total), the small sample size and limited statistical analysis limits the impact of these findings. Jauhiainen et al. found no associations of significance across sub-groups, observing random dispersal in terms of both injury type, location, and proportion of injured and non-injured [13]. Similarly, both Watari et al. [21] and Martin et al. [18] observed seemingly random alignment between sub-groups and injury incidence. Within the scope of current evidence, it seems that RRIs can present with distinctly different movement patterns, and similar kinematic presentations can exist between runners with different injuries and those with no injury.

#### Kinematic Characteristics of Sub-groups

The heterogeneity of kinematic variables measured rendered inter-study comparison of sub-groups challenging. After exclusion of insignificant variables from results, no kinematic characteristics featured consistently across all studies. Only hip adduction and knee abduction were analogous across three studies, and knee flexion across four studies, as kinematic variables by which sub-groups were separated – although these were not aligned to injury classification.

Sub-groups that exhibited reduced hip adduction were observed to have the smallest knee abduction angle [13, 21], suggesting an association between these two variables. Conflictingly, hip adduction was associated with both high [20] and low [13] knee flexion angles. Ankle plantarflexion also featured as a sub-group differentiator across two studies [18, 19]. Although some independently measured variables (for example, tibia inclination in Dingenen et al. [20], hip internal rotation in Watari et al. [21] and foot progression angles in Jauhiainen et al. [13]) may correlate with one another [23, 24], it would not be possible to accurately quantify these correlations in order to conduct a cross-study comparison. However, it must be noted that both Dingenen et al. [20] and Jauhiainen et al. [13] noted similarities in kinematic patterns across different groups, with certain clusters correlating to the same planes of motion. These data suggest sub-groups of runners may exhibit similar gait patterns within ‘sagittal’, ‘frontal’ or ‘transverse’ planes.

Foot-strike pattern proved a common theme across studies. Jauhiainen et al. [13] noted a sub-group of ‘heel-strikers’, whilst Martin et al. [18] noted clustering of runners with a tendency towards a more midfoot strike pattern. Gore [19] identified a five-cluster solution based on foot-strike kinematics alone, and whilst Dingenen et al. [20] did not directly measure foot strike, they noted overstride and foot inclination as a sub-group differentiator.

#### Non-kinematic Characteristics of Sub-groups

**Fig. 2:**
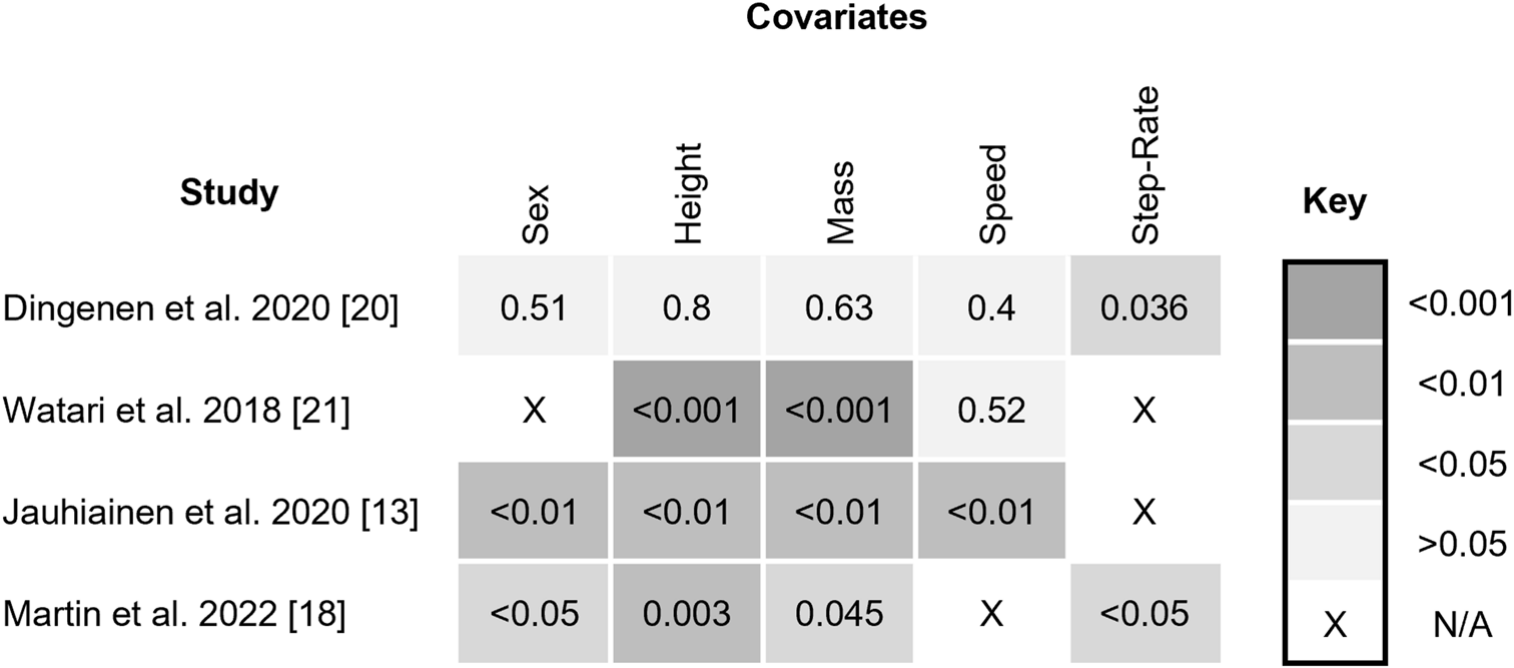
Heatmap depicting the significance of between-group differences, as determined from *p* values (one-way ANOVA)

All primary studies recorded a number of functional and demographic characteristics (Fig. 2). Significant differences in height [13, 21], mass [13, 21], running speed [13] and running step rate [13, 20] were observed between sub-groups in some studies, whilst in others there was seemingly random dispersal.

Differences in height and mass may be explained by the confounder of unequal sex distribution across sub-groups. In general, males displayed greater homogeneity in gait, with the majority clustered into the same kinematic sub-group in the primary studies; 80% [20], 75% [13] and 60% [18] of males were clustered into one sub-group, with the fourth study grouping males and females a priori and identifying no sub-groups within the male cohort [21]. Additionally, 70% females in Jauhiainen et al. [13] were represented within a single sub-group, whilst two sub-groups in Martin et al. [18] were 100% and 83% female. These findings must be interpreted with caution due to relatively small sample sizes; however, they do suggest different variations in gait patterns within male and female runners.

## Discussion

In this scoping review we identified four primary studies and one thesis clustering injured and healthy runners on the basis of kinematic variables. All studies observed the existence of distinct sub-groups based on gait kinematics within the population, although these were all independent of injury status. There was very limited evidence to suggest that sub-groups are associated with injury location. Common distinctions were observed in kinematic and demographic variables between sub-groups.

### RRI Incidence within Sub-groups

Every study observed a random distribution of injury across sub-groups, irrespective of diagnosis or location. These results suggest that injury cannot be predicted by one movement pattern, and therefore there is no single ‘pathological’ gait that separates those with a specific injury from those without. This conclusion indicates that, despite previous research finding a significant difference between injury groups, this does not lead to a conclusion of a specific kinematic pattern being associated with a specific injury pattern. As a result, recommendations made on these conclusions should be viewed with caution.

There was some very limited evidence that injury incidence between sub-groups may differ according to location. In one study, clusters displaying greater tibia and foot inclination exhibited a higher incidence of shin injuries, whilst the cluster which exhibited greater hip adduction and reduced knee flexion were more likely to suffer from hip pathology [20]. However, these findings must be interpreted with caution, due to the small sample size and lack of determination of significance through statistical analysis. Jauhiainen et al. [13] and Gore [19], whose populations were considerably larger, observed random dispersal of both injury type and location across all sub-groups. Based on the available literature, it appears that a specific kinematic gait pattern is not associated with a specific injury, and sub-group does not determine injury prevalence.

A significant limitation of these studies is that they collected and analysed data of an average step calculated from as few as seven [20] steps. This acts on the assumption that this small sample’s average is representative of an individual’s overall movement pattern. However, intra-individual variability in running biomechanics has been well documented in the literature and is influenced by a multitude of factors including fatigue [25], running experience [26, 27], sex [28] and running speed [29]. There is also some, albeit limited, evidence that variability of gait kinematics is associated with prior injury [30]. This information calls into question the validity of the results of the included studies. Future research may control for these factors to investigate this variability to better reflect a runner’s typical gait.

A number of studies have reported the existence of multiple movement patterns within a population of healthy runners [31–34]. Even when matched for demographic factors such as age, height, sex and running speed, distinct homogeneous sub-groups have been identified using 3D kinematic data [35]. The results of our review would suggest that similar results can be observed within an injured population, indicating that runners with the same injury are not defined by one kinematic pattern, and that kinematic differences cannot clearly explain injury aetiology.

By focusing solely on gait kinematics, previous research has been conducted with an underlying assumption that gait kinematics are specific to different injuries. This is not supported by this review, and thus the use of this assessment needs to be undertaken judiciously with a knowledge of the available evidence. Analogous to this alternative perspective is the use of imaging for low back pain. The latest evidence and recommendations refute the standard use of imaging for back pain as a standalone diagnostic tool, due to the lack of sensitivity and specificity [36]. Utilising kinematic analysis of gait to diagnose/assess mechanisms of RRI may be a similar misleading tool when used standalone.

However, although ambiguous, it is apparent that gait kinematics does play a role in injury. Taking into account the available evidence demonstrates the need for future research that adopts a longitudinal, prospective approach to understanding the relationship between gait kinematics and injury, integrating monitoring of training, as well as rehabilitation protocols that focus on the individual rather than the diagnosis.

### Mechanisms for the Existence of Sub-groups

A number of factors may play a role in sub-group determination and the underlying mechanisms behind different kinematic presentations [33, 34]. In the included studies, variables of significance between sub-groups were sex, spatiotemporal measures (running speed, running step rate) and anthropometric factors (height, mass). There is evidence in the literature to support the influence of all these variables on running kinematics; in this way they may provide an explanation for the existence of different sub-groups.

#### Anthropometric Factors

Jauhiainen et al. [13] and Watari et al. [21] noted significant inter-group variability in height and mass, whilst Martin noted differences in mass alone. One explanation for this would be the unequal distribution of sexes between groups, with males being generally taller and heavier. However, Dingenen et al. did not note any significant differences in height and mass across clusters, despite unequal distributions of male/female between the two sub-groups. This is further supported in the literature, with Vincent et al. [37] observing biomechanical differences in sex- and age-matched runners. Body mass index has also been observed to correlate with changes in vertical loading [38, 39] and peak frontal and sagittal plane hip moments [40]. Thus, it is possible that height and mass may influence a runner’s biomechanics independently of sex and in this way contribute to sub-group formation.

Osteometric factors, although not measured in the included studies, have been demonstrated to influence kinematics, and thus may provide an explanation for the existence of different movement patterns. For example, inter-group variability in foot strike patterns and inclination, as noted by four studies [13, 18–20], may be influenced by foot structural differences [41, 42]. Similarly, anterior superior iliac spine width has been shown to be associated with foot-inclination angles, peak hip adduction and ankle dorsiflexion [43], whilst Q-angle is believed to influence tibial internal rotation [44], a significant variable in Dingenen et al.’s study [20]. However, future research is needed to definitively characterise the existence and magnitude of these potential influences on kinematic sub-group formation.

#### Sex-Related Factors

All primary studies noted a sex predisposition to certain kinematic patterns. Jauhiainen et al. [13] identified one sub-group consisting of 79% female runners, who had the greatest hip adduction angles. In the same study, one predominantly male group observed low knee abduction and low hip adduction angles. Another sub-group consisting of entirely female runners [18] exhibited second-greatest peak knee extension moments, whilst predominantly male groups exhibited the lowest [18]. Meanwhile, Dingenen et al. concluded that kinematic differences were entirely sex related, since there were no significant variables to distinguish female sub-groups, apart from hip internal rotation [20].

Sex differences in lower-extremity biomechanics are well documented [14, 15, 45–47]. Overall, these results are consistent with previous studies suggesting that female runners generally exhibit greater frontal plane hip and knee peak angles, as well as reduced sagittal plane peak knee angles [14, 15, 45, 46, 48]. Previous literature has also observed sex differences in sports injury incidence [47], or ‘sex bias’, and hypothesised this to be attributable to kinematic differences. Conversely, it is unlikely that sex explains all sub-group variability. Jauhiainen et al. [13], Dingenen et al. [20] and Martin et al. [18] identified groups containing equal proportions of male and female runners; similarly, studies have observed homogeneous sub-groups of runners within sex-matched populations [49–51].

Evidence from the included studies observes a sex predisposition to certain kinematic patterns. If future research were to identify multiple kinematic patterns contributing to injury, it is likely that one may have a sex predisposition, but others may not, reflecting the likelihood of multiple aetiological frameworks for RRIs. However, further exploration is needed in this area to draw evidence-based conclusions and direct clinical and rehabilitation practice.

#### Spatiotemporal Measures

Both running speed [13] and step rate [19, 20] varied significantly between sub-groups in three studies. Running speed has been demonstrated to influence hip, knee and ankle kinematics in a linear fashion [52]. This may explain some of the differences between fast and slow groups [13] and highlights the need to control for this variable in future research. Where running step rate varied, foot inclination angles [20], time in stance phase [18] and foot-strike patterns [19] also differed significantly between sub-groups. Given the established relationship between these variables [53–56], there is a compelling argument for the role of step rate in kinematic sub-group differentiation.

#### Muscle Strength

Muscle strength was not measured in any of the studies; however, it is an important factor to consider when hypothesising between-group kinematic variability. Most notably, increased abductor strength may affect knee-flexion and internal-rotation angles [57–59], whilst hamstring strengthening may impact ankle plantarflexion, thus altering ankle and foot kinematics [60]. Observing differences across clusters should therefore be considered for future research, in order to understand whether muscle strength varies between running sub-groups.

### Clinical Application

Taking into account the available evidence, it is unlikely that one movement pattern can explain a given RRI, or that kinematic factors alone are responsible for injury aetiology. It is more likely that multiple dysfunctional movement patterns exist, which may only lead to injury with sufficient exposure to external training loads. Similarly, training errors may only lead to recurrent injury in the presence of certain kinematic risk factors. In this way, abnormal loading patterns, in the context of external factors such as training load or tissue strength, may determine injury development, and thus explain why there was apparent random dispersal of injuries across sub-groups.

Based on limited evidence for the association of injury location with kinematic sub-groups, it is possible that certain movement patterns may predispose to overloading of a certain body region rather than specific injury. Future research should be guided by anatomical planes or location, rather than diagnosis, to further explore this hypothesis.

Clinicians should take all the available evidence (including previous research) into account when assessing an RRI, and especially consider dysfunctional kinematics in the context of recurrent injury, and where there are no obvious training errors. However, as of yet, there are insufficient data to link specific ‘pathological’ gaits to specific injuries. Therefore, we recommend that athletes be managed on an individual basis, and ideally monitored prior to injury to identify any kinematic changes that may predispose to tissue overload.

### Limitations of the Literature

Two of the studies [13, 21] extracted kinematic data from the same existing database of runners (Running Injury Clinic, University of Calgary). Although Jauhiainen et al. [13] evaluated a larger cohort (*n* = 291) compared with Watari et al. [21] (*n* = 110), it was not possible to ascertain the extent of overlap between the two populations. This reduces the applicability and representativeness to the running population and possibly the significance of the results.

One limitation that must be noted when comparing clusters is the heterogeneity in the populations studied. Only Watari et al. [21] matched for sex, and no included papers matched for any other significant demographic factors, such as height, mass or running speed. Studies have demonstrated the importance of data matching on kinetic and kinematic variables in runners [48]. As significant differences in these confounders were seen between clusters in the included studies, we cannot conclusively determine whether the between-group kinematic differences observed are a result of these potential covariates, or simply a characteristic of that group. As such, there is significant scope for future research in this area.

It is also difficult to distinguish causality from compensatory mechanisms for injury. Different gait patterns may reflect different methods of load distribution, resulting in injury, or they may be the result of alterations due to pain response or neuromuscular dysfunction in response to existing pathology. For example, differences in structure-specific loading in frontal or sagittal planes may explain the increase in hip and shin injury incidence, respectively [20, 34, 61, 62], or result from compensation for pain or weakness [63, 64]. Therefore, without further prospective evidence we cannot establish whether specific biomechanical presentations can represent the specific RRI, or whether the same RRIs present with the same gait pattern.

## Conclusion

There is evidence in the literature for the presence of distinct sub-groups exhibiting homogeneous kinematic gait patterns within a population of injured and healthy runners. We found no robust evidence to infer an association between sub-groups and specific RRIs, and limited evidence to suggest an association with injury location. Notwithstanding, the existence of kinematic variability within injured populations refutes the connection of certain gait patterns to certain injuries. In this respect, it is likely that multiple mechanisms underpin RRI aetiology, the understanding of which should be the focus of future research as well as rehabilitation protocols that focus on the individual rather than the diagnosis.

### Scope for Future Research

This review has revealed a large scope for future investigation, to better understand and quantify variability in running gait and its role in injury aetiology. Research needs to include large-scale, longitudinal studies that acknowledge the natural variability in injured populations and group individuals accordingly. It is important to match study populations as closely as possible according to sex, height and mass to reduce the influence of these confounders on inter-group kinematic variability. Longitudinal studies following a population of prospectively injured participants over time will enable us to determine high-risk gait patterns and better understand the complex relationship between running gait and injury pathogenesis.

### Supplementary Information

Below is the link to the electronic supplementary material.

Supplementary file1 (PDF 259 KB)
